# Effects of parental short message service reminders on infant immunisation coverage, timeliness and barriers in Nigeria: A quasi-experimental study

**DOI:** 10.51866/oa.744

**Published:** 2025-01-21

**Authors:** Hamina Dathini, Siti Khuzaimah Ahmad Sharoni, Kever Robert

**Affiliations:** 1 MSc (Nursing), PhD (Community Health) Centre for Nursing Studies, Faculty of Health Sciences, Universiti Teknologi MARA, Puncak Alam, Malaysia. Email: sitik123@uitm.edu.my; 2 N.Sc, MSc (Nursing), Centre for Nursing Studies, Faculty of Health Sciences, Universiti Teknologi MARA, Puncak Alam, Malaysia.; 3 MSc, PhD (Community Health Nursing), Department of Nursing Science, Faculty of Allied Health Sciences, University of Maiduguri, Maiduguri, Nigeria.

**Keywords:** Infant, Text Messaging, Parents, Vaccination Coverage, Nigeria

## Abstract

**Introduction:**

This study aimed to determine the effects of parental short message service (SMS) reminders on infant immunisation coverage, timeliness and barriers and evaluate the effects of sex on immunisation outcomes in Maiduguri, Borno State, Nigeria.

**Methods:**

This two-arm quasi-experimental study was conducted in two primary healthcare facilities selected using simple random sampling with opaque envelopes. A total of 524 participants were enrolled using purposive sampling. Data were statistically analysed using the Statistical Package for the Social Sciences version 28 with repeated-measures logistic regression analysis, the Z test for Poisson rates and the Wilcoxon signed-rank test.

**Results:**

The parental SMS reminders significantly improved the immunisation coverage, as reported by 69 (26.3%) (P=0.001, 95% confidence interval [CI] = 12.2-40.5), 117 (44.7%) (P=0.001, 95% CI=32.6-56.9) and 116 (44.3%) participants (P=0.001, 95% CI=34.2-54.4) for the 6th-, 10th- and 14th-week schedules, respectively. Compared to mothers’ involvement, fathers’ involvement did not significantly affect the immunisation coverage (B=0.158, P=0.311, 95% CI=-0.148–0.464). With regard to the immunisation timeliness, the parental SMS reminders yielded a significant effect for the 6th-, 10th- and 14th-week schedules (P=0.001, 95% CI=25.9-46.7; P=0.001, 95% CI=24.2-43.0; and P=0.001, 95% CI=21.1–36.9, respectively). Compared to mothers’ involvement, fathers’ involvement significantly influenced the immunisation timeliness (B=0.298, P=0.038, 95% CI=0.016–0.579). Lastly, the parental SMS reminders significantly reduced the barriers to immunisation, with a P-value of 0.001.

**Conclusion:**

Parental SMS reminders can significantly improve immunisation outcomes in Nigeria.

## Introduction

Despite the well-documented evidence on the importance of vaccines, immunisation coverage in developing countries is low.^1^ The international benchmark for immunisation coverage is 90%; however, as of 2018, only 25% of infants in Nigeria were immunised, among whom only 45% received their vaccines on time.^2^ By 2021, this coverage dropped to 23%.^1^ Such decline is attributed to the high rate of non-adherence to recommended vaccines, driven by barriers such as forgetfulness, a lack of knowledge about vaccines, myths and misconceptions, negative previous immunisation experiences (e.g. adverse or side effects) and family and social influences.^3^ This study focuses on the influences of forgetfulness and family and social structures on immunisation outcomes.

Poor immunisation coverage and timeliness lead to increased vaccine-preventable deaths.^4^ According to evidence, over 700,000 infant deaths are due to vaccine-preventable diseases (VPDs), with 99% of such deaths occurring in developing countries.^5,6^ Furthermore, Africa and Asia alone contribute about 70% of vaccine-preventable deaths, of which 44% happen in Africa.^7^ Hence, infants born in African countries with poor immunisation coverage, such as Nigeria, are about 34 times more likely to die of VPDs than those born in countries with good immunisation coverage.^7^ In this context, the infant mortality rate in Nigeria exceeds 100 deaths per 1000 live births,^5^ while the under-5 mortality rate due to VPDs stands at 22%.^4^

Mobile health (mHealth) interventions have been reported to improve the healthcare service coverage of public health interventions, dissemination of information and promotion of positive healthcare-seeking behaviour.^2^ The growing shift in attention to the use of mHealth interventions is attributed majorly to the vast distribution of mobile phones and the convenience that comes with their use.^8^ Different studies have reported the effectiveness of mHealth interventions in improving immunisation coverage and timeliness^1,9-11^; however, they have not been tested in Borno State, Nigeria. Additionally, the systematic review conducted by Dathini et al.^12^ showed that all studies conducted in Nigeria focused on women, with no male involvement.^1,9-11^ This is despite the fact that Nigeria and most developing countries operate a patriarchal system.^13^

Given the abovementioned context, this study aimed to determine the effects of parental SMS reminders on infant immunisation coverage, timeliness and barriers in Borno State, Nigeria.

## Methods

### Design and population

This study adopted a two-arm parallel quasi-experimental design targeting the parents of infants aged <4 weeks.

### Inclusion criteria

Participants were recruited in the study when they had infants aged <4 weeks, were a mother or a father with an active mobile phone and had no intention to relocate within 6 months. For twin babies, the index child was considered the youngest.

### Exclusion criteria

Participants who were aged <18 years and could not read messages in English or Hausa or at least find someone within their household to read and interpret the messages were excluded.

### Setting

This study was conducted in Maiduguri, Borno State, Nigeria. Borno State is one of the 36 states in Nigeria. It has a population of 5.86 million people and a fertility rate of 5.2, according to the 2018 National Demographic Health Survey.^14,15^ Of this population, evidence shows that 2.6 million are active users of mobile phones.^16^ This is considered high when children and other minors are removed from the population. Additionally, 74.4% of Nigerians have active mobile phone numbers, which is expected to rise further because of dependence on technology.^4^

### Intervention

The study intervention was parental SMS reminders. These reminders were sent to participants based on their preferred language (English or Hausa) twice on day 3 and once during the immunisation due date for the 6^th^-, 10^th^- and 14^th^-week schedules. The SHOTS survey (http://www.shotsurvey.org/) was administered at enrolment and endline to explore the barriers to immunisation. Conversely, the control group continued with their standard care practice.

### Outcome measures

The primary outcomes of this study were the coverage and timeliness of immunisation. Immunisation coverage was assessed based on the percentage of vaccines infants received regardless of the administration date of antigens. Immunisation was considered timely when it was administered ±3 days from the scheduled due date.^1^ The secondary outcome was the barriers to immunisation.

### Sample size calculation

For the calculation of the sample size and attrition rate, the formula from Sakpal^17^ was used, along with the results from the study by Ibraheem et al.^1^: n=[(Z_α/2_ + Z_β_)^2^ ×{(p1 (1-p1)+(p2 (1-p2))}]/(p1-p2)^2^. An attrition rate of 20% was estimated, resulting in a total sample size of 524 participants, with 262 participants per group.

### Randomisation

Facilities were randomised at the level of intervention assignment using the simple random sampling technique involving opaque envelopes, while participants were recruited using the purposive sampling technique.

### Sampling method and allocation concealment

Simple random sampling using opaque envelopes was applied to select healthcare facilities. Accordingly, all healthcare facilities were listed on a separate paper, concealed in sealed opaque envelopes and shuffled thoroughly. Thereafter, two individuals coded as A (intervention group) and B (control group) picked one envelope each. Conversely, purposive sampling was utilised to enrol participants. A total of 131 men and women were enrolled in the intervention group and 262 men and women in the control group.

### Blinding

The study was a single-blind study, with only the healthcare workers being blinded.

### Statistical analysis

Data were analysed using the Statistical Package for the Social Sciences version 28 (IBM SPSS, 2022, USA). For the socio-demographic data, frequencies, percentages, means, standard deviations and interquartile ranges were used. Additionally, repeated-measures logistic regression was utilised to determine the effects of the intervention at the 6^th^, 10^th^ and 14^th^ weeks, while the Wald chi-square test was employed to test the effects of the intervention between the father and mother sub-groups. The Z test for Poisson rates was used to compare the coverage and timeliness of immunisation across the intervention schedules, while the Wilcoxon signed-rank test was conducted to determine the barriers to immunisation. The results were considered significant when the P-value was <0.05 at a 95% confidence interval (CI).

## Results

### Participant recruitment and enrolment

**Figure 1:**
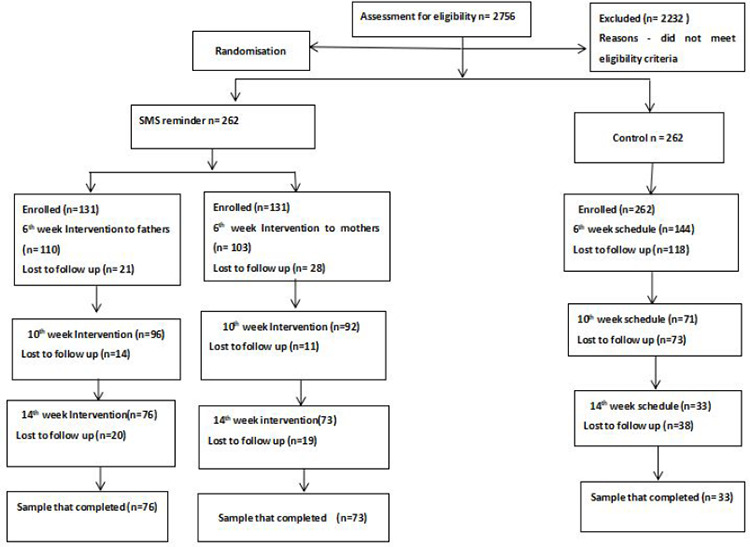
Flowchart of participant selection.

Participant recruitment began on the 28th of July and ended on the 6 th of October 2023. A total of 2756 participants were assessed for eligibility, among whom 524 were finally included in the study, with 262 participants in each group. The flowchart of participant selection is shown in Figure 1.

As shown in Table 1, the mean age of the participants was 29±7 years. The majority were Muslims (n=499, 95.2%) and completed senior secondary school (n=191, 36.5%). Additionally, the most common place of delivery was government facilities (n=328, 62.5%), and the reported mean income was 284,047 naira.

**Table 1:** Socio-demographic data (N=524).

Variable	n	%
**Age, year**		
Mean (SD)	29±7	
**Religion**		
Muslim	499	95.2
Christian	25	4.8
**Educational level**		
No formal education	187	35.7
Primary education	49	9.4
Secondary education	191	36.5
Post-secondary education	68	12.9
University education	27	5.2
**Income**		
Mean	₦284,047^*^	
Place of delivery		
Government facility	328	62.5
Private facility	176	33.6
Home	20	3.8

* ₦1607.5=US $1 (12/8/2024)

### Effects of the parental SMS reminders on the immunisation coverage

Table 2 reveals that the intervention improved the immunisation coverage, as reported by 69 (26.3%) (P=0.001, 95% CI= 12.2-40.5) 117 (44.7%) (P=0.001, 95% CI=32.6-56.9) and 116 (44.3%) participants (P=0.001, 95% CI=34.2-54.4) for the 6^th^-, 10^th^- and 14^th^-week immunisation schedules, respectively, compared to the control. Additionally, male involvement led to an improvement in the immunisation coverage across the schedules, although the effect was not significant.

**Table 2:** Effects of Short Message Services (SMS) reminders on immunisation coverage.

	Group		Diff.	95% CI for diff.	Z-value	P-value
	Control (n=262)	SMS reminder (n=262)				
Coverage						
6^th^ week	144 (55.0)	213 (81.3)	69 (26.3)	12.2-40.5	3.65	0.001
10^th^ week	71 (27.1)	188 (71.8)	117 (44.7)	32.6-56.9	7.27	0.001
14^th^ week	33 (12.6)	149 (56.9)	116 (44.3)	34.2-54.4	8.60	0.001
	Parent					
	Father (n=131)	Mother (n=131)
**Coverage**						
6^th^ week	110 (84.0)	103 (78.6)	7 (5.4)	-9.8-18.9	0.48	0.631
10^th^ week	96 (73.4)	92 (70.2)	4 (3.2)	-16.5-27.2	0.29	0.770
14^th^ week	76 (58.0)	73 (55.7)	3 (2.3)	-16.0-20.6	0.25	0.806

### Effects of sex on the immunisation coverage

The intervention improved the immunisation coverage, regardless of sex (B=0.77, P=0.001, 95% CI=0.562-0.987). However, compared to mothers’ involvement, fathers’ involvement did not significantly improve the immunisation coverage (B=0.158, P=0.311, 95% CI=-0.148-0.464), as shown in Table 3.

**Table 3:** Effects of sex on immunisation coverage.

Source	Type III		
	Wald chi-square	Df	Sig.
(Intercept) Sex	119.702 1.025	1 1	0.000 0.311

Parameter estimate							
Parameter	B	Std. error	95% Wald confidence interval		Hypothesis test		
			Lower	Upper	Wald chi-square	Df	Sig.
(Intercept)	0.774	0.1085	0.562	0.987	50.903	1	0.001
Father	0.158	0.1560	-0.148	0.464	1.025	1	0.311
Mother (RC)							

RC: reference category

### Effects of the parental SMS reminders on the immunisation timeliness

Table 4 shows that the intervention significantly improved the immunisation timeliness, as reported by 95 (36.3%) (P=0.001, 95% CI=25.9-46.7), 88 (33.5%) (P=0.001, 95% CI=24.2 - 43.0) and 76 (29%) participants (P=0.001, 95% CI=21.1–36.9) for the 6^th^-, 10^th^- and 14^th^-week schedules, respectively. Although the intervention improved the immunisation coverage in the father sub-group, the effect was not significant across all three schedules.

With regards to the effects of sex on timeliness, the parental SMS reminders significantly improved the immunisation timeliness, regardless of sex (B=0.313, P=0.002, 95% CI=0.113 - 0.513). However, fathers’ involvement significantly affected the immunisation timeliness (B=0.298, P=0.038, 95% CI=0.016-0.579).

**Table 4:** Effects of SMS reminders and sex on immunisation timeliness.

	Group					
	Control (n=262)	SMS reminder (n=262)	Diff.	95% CI for diff.	Z-value	P-value
Timeliness						
6^th^ week	49 (18.7)	144 (55.0)	95 (36.3)	25.9-46.7	6.84	0.001
10^th^ week	35 (13.4)	123 (46.9)	88 (33.5)	24.2-43.0	7.00	0.001
14^th^ week	18 (6.9)	94 (35.9)	76 (29)	21.1-36.9	7.18	0.001
	**Parent**					
	**Father (n=131)**	**Mother (n=131)**				
Timeliness						
6^th^ week	80 (61.1)	64 (48.9)	16 (12.2)	-5.7-30.2	1.33	0.182
10^th^ week	65 (49.6)	58 (44.3)	7 (5.3)	-11.2-21.9	0.63	0.528
14^th^ week	50 (38.2)	44 (33.6)	16 (4.6)	-9.9-19.1	0.62	0.536

Source	Type III		
	Wald chi-square	Df	Sig.
(Intercept) Sex	5.228 4.300	1 1	0.022 0.038

Parameter estimate							
Parameter	B	Std. error	95% Wald confidence interval		Hypothesis test		
			Lower	Upper	Wald chi-square	Df	Sig.
(Intercept)	0.313	0.1021	0.113	0.513	9.391	1	0.002
Father	0.298	0.1436	0.016	0.579	4.300	1	0.038
Mother (RC)							

RC: reference category

### Effects of the parental SMS reminders on the barriers to immunisation

Table 5 shows that the mean total score was 81.52 for the positive rank and 59.84 for the negative rank. The access, concern and importance sub-scales had P-values of 0.001, 0.157 and 0.022, respectively. The post-test effect, when compared to the pre-test effect, was significant at a P-value of 0.001.

**Table 5:** Effects of SMS reminders on barriers to immunisation.

		Mean score	Sum of score
Access 2 - access	Negative rank	48.42^a^	1452.50
	Positive rank	78.26^b^	8843.50
Concern 2 - concern	Negative rank	60.75^d^	3827.00
	Positive rank	72.63'	5084.00
Importance 2 - importance	Negative rank	71.33^g^	4708.00
	Positive rank	51.19^h^	2918.00
Total score 2 - total score 1	Negative rank	59.84^j^	3052.00
	Positive rank	81.52^k^	7826.00

Test statistic^a^				
	Access 2 – access	Concern 2 – concern	Importance 2 – importance	Total score 2 – total score 1
Z	-7.453^b^	-1.414^b^	-2.282^c^	-4.618^b^
Asymp. sig. (two-tailed)	0.000	0.157	0.022	0.001

a Wilcoxon signed-rank test

## Discussion

To the researchers’ knowledge, this study provides the first set of knowledge regarding the influence of sex on immunisation outcomes using parental message reminders. The study revealed that the mean age of the participants was 29±7 years, with the majority being multiparous women. The participants were expected to have substantial knowledge of immunisation despite their low educational level. Additionally, the fact that the most common place of delivery was government facilities could be related to their policies of providing free maternal and child healthcare services.

Adequate immunisation coverage is a cost-effective strategy for preventing infant and childhood morbidity and mortality.^18^ This underpins the importance of vaccines in boosting children’s immunity and preventing these undesired outcomes.^19^ Accordingly, this study determined the effects of parental SMS reminders on immunisation coverage in Maiduguri, Borno State. The results revealed that the parental SMS reminders significantly improved the immunisation coverage among infants across the 6^th^-, 10^th^- and 14^th^-week immunisation schedules. This improved immunisation coverage is consistent with previous reports in Nigeria and Canada.^1,20^ This implies that the intervention is effective in improving immunisation coverage in developing and developed countries. According to evidence, parents are essential stakeholders in the success of infant immunisation programmes by ensuring that infants receive all the needed vaccines.^21^ Despite parents’ critical role, they could be constrained by forgetfulness.^20^ This forgetfulness is often linked to a busy work life or a lack of awareness of the scheduled date.^22^

With regards to the influence of sex, this study showed that compared to mothers’ involvement, fathers’ involvement improved the immunisation coverage across the 6^th^-, 10^th^- and 14^th^-week immunisation schedules. However, the effect was not significant. This finding is contrary to that of Gelagay et al.,^23^ wherein male involvement in immunisation was positively associated with improved immunisation coverage. Gelagay et al.^23^ conducted their study in Ethiopia, which has a different demographic profile compared to Borno. This difference could account for the disparity seen in this study.

The intervention had a significant overall effect on the timeliness of immunisation, which is in line with previous reports.^4,24^ This demonstrates the positive effect of parental SMS reminders on immunisation timeliness. Timely vaccination is increasingly seen as an essential indicator for preventing the needless death of children from VPDs.^4^ However, evidence suggests that forgetfulness could hamper immunisation timeliness.^3^ Reminding parents through SMS could be attributed to the significant improvement noted in the present study.

In terms of the effects of sex, this study showed that fathers’ involvement had an overall positive effect on the immunisation timeliness compared to mothers’ involvement. According to evidence, the decision to seek care and pay for healthcare services is largely seen as men’s responsibility.^25^ Hence, the inability of women to make independent healthcare decisions often results in delays in seeking healthcare services.^26^ This is primarily attributed to the inability to pay for direct and indirect costs. The timely uptake of immunisation in the father sub-group of this study is a reflection of timely decision-making and the provision of the needed finances.^27^

In this study, the parental SMS reminders were found to significantly reduce the barriers to immunisation. Similarly, a previous study reported that SMS reminders reduced missed immunisation appointments.^20^ Forgetfulness and long walking distances greater than 30 minutes, along with poverty, are also identified as barriers.^20,28^ Timely immunisation reminders provided to parents of infants could afford them more time to arrange for transportation funds.

Over the past years, digital technologies such as real-time monitoring platforms (e.g. RapidPro, Open Data Kit and District Health Information System 2) have been deployed across different stages of the vaccine supply chain and delivery to promote immunisation outcomes in Nigeria.^29^ Although their use has substantially improved immunisation outcomes, evidence has consistently shown that immunisation coverage in Nigeria remains low and that the prevalence of VPDs is increasing.^4,5^ It is therefore interesting to observe that simple technology such as parental SMS reminders can significantly improve immunisation outcomes.

## Limitations

This study focused only on the 6^th^-, 10^th^- and 14^th^-week immunisation schedules and parents with mobile phones, which may influence the generalisability of the findings.

## Conclusion

Parental SMS reminders significantly affected the immunisation coverage, timeliness and barriers. Fathers’ involvement also yielded a significant effect on the timeliness of immunisation. Although this study recorded significant improvements in the immunisation outcomes, there is still a need for further studies to cover the entire childhood immunisation schedule and upscale the intervention to different parts of the country, especially those that have good mobile phone coverage.

## Data sharing statement

The dataset used in this study can be accessed for ethical use upon request from the corresponding author.

**How does this paper make a difference in general practice?**

- This study provides substantial evidence that mobile health interventions can be used to improve immunisation coverage and timeliness in Nigeria.
- The results emphasise the importance of male involvement in immunisation programmes.
- Parental short message service reminders can reduce the barriers to immunisation parents face.
